# Investigation of the excitations of plasmons and surface exciton polaritons in monoclinic gadolinium sesquioxide by electron energy-loss spectroscopy and plasmon spectroscopic imaging

**DOI:** 10.1039/d2ra00737a

**Published:** 2022-04-04

**Authors:** Sz-Chian Liou, Vladimir P. Oleshko, W. Chun-Hsin Kuo, Tan-Ju Yang, Guo-Jiun Shu

**Affiliations:** Advanced Imaging & Microscopy Laboratory, Maryland NanoCenter, Institute for Research in Electronics and Applied Physics, University of Maryland College Park MD 20742 USA scliou@protonmail.com; Material Measurement Laboratory, National Institute of Standards and Technology Gaithersburg MD 20899 USA; Materials Characterization Facility, Texas A&M University College Station TX 77845 USA; Department of Materials and Mineral Resources Engineering, National Taipei University of Technology Taipei 10608 Taiwan gjshu@mail.ntut.edu.tw; Institute of Mineral Resources Engineering, National Taipei University of Technology Taipei 10608 Taiwan; Taiwan Consortium of Emergent Crystalline Materials, Ministry of Science and Technology Taipei 10622 Taiwan

## Abstract

The monoclinic gadolinium sesquioxide (denoted as m-Gd_2_O_3_) with its lower crystal symmetry exhibits larger dielectric permittivity (*κ*) than the cubic Gd_2_O_3_ (denoted as c-Gd_2_O_3_). Recently, a few nanometers thick m-Gd_2_O_3_ thin film has been successfully epitaxially grown on a GaN substrate as a promising candidate gate oxide in metal-oxide-semiconductor field-effect transistors (MOSFETs). Thus, it is important to understand the electronic excitations in m-Gd_2_O_3_ and investigate them by electron energy loss spectroscopy (EELS) performed with aloof electron beams and electron diffraction to gain the spatial and momentum resolutions. In this study, using scanning transmission electron microscopy combined with EELS (STEM-EELS) in the aloof electron beam setup, we observed low-loss spectral features at 13 eV and 14.5 eV at the specimen edge in a grazing incidence and the material interior, which can be interpreted as a surface plasmon (SP) and a volume plasmon (VP), respectively. Surface exciton polaritons (SEPs), which represents surface resonances associated with excitonic onsets above the bandgap, were also observed at about 7, 10.2, and 36 eV energy loss. Their surface excitation character was confirmed by energy-filtered transmission electron microscopy spectrum imaging (EFTEM-SI) and using relativistic energy *versus*-momentum (*E*–*k*) map calculations. The momentum (*q*)-dependent EELS indicates that the SEP features near the bandgap represented a function of *q* and revealed a nondispersive behavior for VP and SEP at 36 eV. The oscillator strengths for VP and SEP at 36 eV dropped at different *q* values along with different *q* directions, revealing the anisotropic electronic structures of m-Gd_2_O_3_.

## Abstract 2

The electronic excitations in m-Gd_2_O_3_ were systematically studied by low-loss EELS in scanning transmission electron microscopy (STEM) mode and electron diffraction mode to gain both the spatial and momentum (*q*) resolutions.

## Graphical abstract

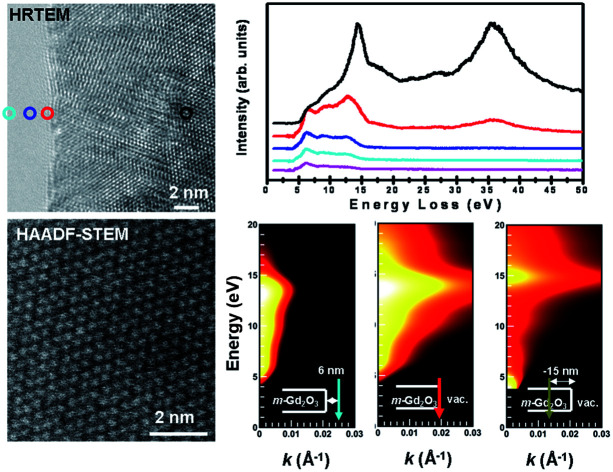

## Introduction

Gadolinium oxide (Gd_2_O_3_) with a large bandgap of about 5.3 eV (ref. 1) and high-permittivity constant (*ε*_r_ or *k* = 14 to 20)^2,3^ has attracted much attention in the microelectronic community as a potential high-*k* dielectric material for applications in complementary metal-oxide-semiconductor (CMOS) and metal-oxide-semiconductor field-effect transistors (MOSFETs). The *k* values ranging from 14 to 20 suggest a ∼4 nm thickness to satisfy the requirement of a 1 nm equivalent oxide thickness (EOT). Here, the EOT is equal to *t*_high-*κ*_(*κ*_SiO_2__/*κ*_high-*κ*_), where *t*_high-*κ*_ is the thickness of high-*κ* dielectrics and *κ*_SiO_2__ = 3.9 is the dielectric constant of SiO_2_. The growth of Gd_2_O_3_ films on various semiconductor substrates with abrupt interfaces and single domain structure is required to prevent the leakage and decrease the capacitance induced by grain boundaries and interfacial layers formed under annealing during the fabrication of CMOS and MOSFET devices. The cubic phase Gd_2_O_3_ (c-Gd_2_O_3_, space group *Ia*3̄) film with a single domain structure has been successfully epitaxially grown on GaAs,^2,4^ Si,^3,5^ and Ge^6^ substrates when the film thickness was less than 5 nm. However, after increasing the film thickness above 8 nm, depending on the substrates, to improve the gate capacitance, the epitaxial c-Gd_2_O_3_ films tend to transform from the cubic phase to the monoclinic phase.^6,7^ Most recently, monoclinic phase Gd_2_O_3_ (m-Gd_2_O_3_, space group of *C*2/*m*) was stabilized in the thin films and the bulk form *via* epitaxial growth on a GaN substrate^8^ and by doping during the fabrication processes.^9^ Most importantly, the *k* value of m-Gd_2_O_3_ was higher than 20 due to its crystal symmetry,^10^ which leads to an essential improvement of the EOT values. Therefore, a deeper understanding of the electronic band structure, including bandgap and band offset for m-Gd_2_O_3_ film/semiconductor heterostructures, becomes crucial for further CMOS and MOSFET microelectronic technology applications.

It was found that 10 nm-thick Gd_2_O_3_ films minimize the effect of moisture absorption on the electrical performance.^8,11^ However, technologies beyond the 16 nm CMOS require the combination of high-carrier-mobility semiconductors and high *k* gate dielectric for further reducing the EOT to less than 1 nm.^11^ The thickness of the m-Gd_2_O_3_ films on various semiconductor substrates is restricted in the range of 4 to 8 nm when considering an EOT value less than 1 nm. Furthermore, the restricted thickness makes it even more challenging to investigate the electronic excitations in m-Gd_2_O_3_ thin films and exclude the effects of interface plasmons generated in the heterostructures.^12–14^ Thus, it is first necessary to understand the electronic excitations in bulk m-Gd_2_O_3_ material.

The information about electronic excitations, particularly concerning transitions between valence and conduction bands in m-Gd_2_O_3_, studied by electron energy loss spectroscopy (EELS) and theoretical band structure calculations, are lacking in the literature in contrast to c-Gd_2_O_3_.^14–16^ Shu *et al.* investigated the core-level electronic excitations of c- and m-Gd_2_O_3_ by EELS.^14^ No distinguishable difference for the Gd N_4,5_-edge excited from the occupied 4d orbital states to the unoccupied 4f orbital states was observed in both c- and m-Gd_2_O_3_. In contrast, the O K-edge exhibited distinct differences in both the spectral features and energies due to the different Gd and O coordination and Gd–O bond lengths.^14^ Furthermore, Shu *et al.* also reported some differences in the low-loss region between c- and m-Gd_2_O_3_, however, without detailed analysis for m-Gd_2_O_3_.^14^ Besides, Gd_2_O_3_ has a larger bandgap of about 5.3 eV and the interband transitions generating weakly bound delocalized excitons presumably of the Wannier–Mott type readily build up collective resonances at the material surface. Furthermore, the surface resonances associated with transverse excitonic onsets could be assigned to surface exciton polaritons (SEPs).^13,14,17^ Indeed, the surface-related excitations will dominate if the material thickness is continuously decreased. Thus, the SEPs modes would be much more easily observed in the thinner thickness of m-Gd_2_O_3_.

In this work, the electronic excitations in m-Gd_2_O_3_ were systematically studied by low-loss EELS with nonpenetrating incident electron beam (aloof excitation) in scanning transmission electron microscopy (STEM) mode and electron diffraction mode to gain both spatial and momentum (*q*) resolution.

## Experimental

c-Gd_2_O_3_ powders (99.99% purity, ACROS*) were used as the starting material, pressed as a pellet, and then calcined in air at a temperature above 1200 °C to form the m-Gd_2_O_3_.^9,14^ A Bruker D8 X-ray diffractometer was used to determine the phase purity and crystallinity for the synthesized m-Gd_2_O_3_ pellets. TEM specimens were prepared using a tripod polishing technique and then thinned by Ar^+^ ion milling operated at 5 kV until a hole formed, and then operated at 0.3 kV to remove the surface amorphous layers.^13,14^ Microstructures and electronic excitations were examined using a Thermo Fisher Themis 300 [(S)TEM] equipped with an electron monochromator and Gatan Image Filter (GIF, model Quantum 965) operated at 200 kV. The energy resolution with electron monochromator was 0.2 eV throughout the STEM-EELS experiments. The momentum (*q*)-dependent EELS experiments were carried out in the diffraction mode with *q* resolution of 0.015 Å^−1^. Real-space energy-filtered TEM (EFTEM) spectrum-imaging (EFTEM-SI) with a tunable energy-selection slit was performed on a JEOL 2100F (S)TEM equipped with a Gatan Image Filter (GIF, Tridiem 863), which was operated at 197 kV accelerating voltage. The single-scattering EELS distributions were derived by deconvolution from the raw EELS data, which was performed by removing the zero-loss peak either by fitting a pre-measured zero-loss peak from vacuum or removing plural scattering with the Fourier-log method. Then, the subsequent Kramers–Krönig analysis (KKA) was conducted using a DigitalMicrograph software package (Gatan Microscopy Suite, Gatan-AMETEK) as described elsewhere.^18–20^ The scattering probabilities of energy *versus*-momentum (*E*–*k* maps) and aloof STEM-EEL spectra as a function of impact parameter were calculated by Kröger's equation^21^ and equations within ref. 22 and 23 in MATLAB scripts. The dielectric data of m-Gd_2_O_3_ was taken from the KKA result.

## Results and discussion

**Fig. 1:**
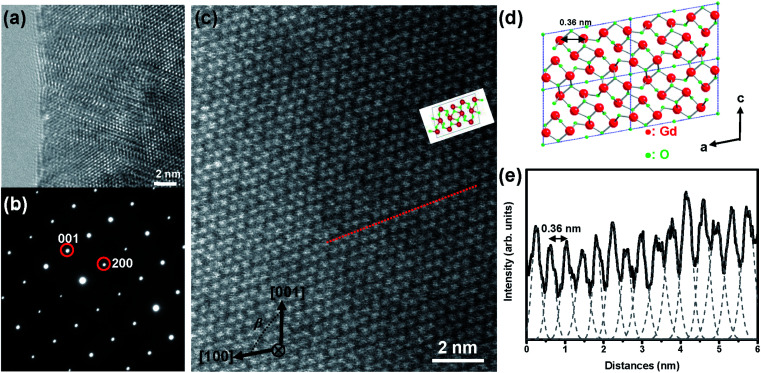
(a) Typical HRTEM image of m-Gd_2_O_3_ recorded at the edge of a m-Gd_2_O_3_ grain. (b) Corresponding SAED pattern at [010] zone axis. (c) High-resolution HAADF STEM image of m-Gd_2_O_3_ recorded along [010] projection. (d) The atomistic model of the m-Gd_2_O_3_ structure in the same orientation with Gd atoms in red and O atoms in green. (e) HAADF STEM intensity profile measured along the red dot line in (c).

Both phase purity and crystallinity of the synthesized m-Gd_2_O_3_ were confirmed by X-ray diffraction (XRD) in the previous report.^14^ Because the *β* angle between the (100) and (001) planes for m-Gd_2_O_3_ is ∼100.13°, it is essential to obtain electron diffraction (ED) patterns and related TEM and STEM images along the [010] orientation, which displays this characteristic *β* angle. Fig. 1(a) shows a representative high-resolution TEM (HRTEM) image of m-Gd_2_O_3_ recorded from the edge of the grain with the size of a few μm. It is a clean edge without any amorphous or damaged area caused by Ar^+^ bombardment. Fig. 1(b) presents the corresponding selected-area electron diffraction (SAED) pattern of m-Gd_2_O_3_ collected along the [010] zone axis from the area shown in Fig. 1(a). The *d*-spacings of the (200) and (001) indexed diffraction spots nearest to the central transmitted spot were 0.67 nm and 0.851 nm, respectively, which is in agreement with the earlier reported XRD data for m-Gd_2_O_3_.^14^ The angle between the two oriented axes was measured at about 100°, which is close to the expected value of the *β* angle for m-Gd_2_O_3_. Furthermore, to examine the material structure at the atomic scale, the high-resolution high-angle annular dark-field (HAADF) STEM imaging of m-Gd_2_O_3_ was performed along the [010] projection [Fig. 1(c)]. The contrast of the HAADF STEM images usually follows the atomic number (*Z*) dependence, *Z*^*n*^, where *n* is usually between 1.3 and 2, the so-called *Z*-contrast.^24^ Although the Gd and O atomic columns along the [010] zone axis are well-separated from each other [see illustrated atomic structure in Fig. 1(d)], it is hard to observe a clear contrast is generated from the O atomic columns due to the large atomic number difference between Gd (*Z* = 64) and O (*Z* = 8). Thus, the bright dots in Fig. 1(c) represent the Gd atomic columns, and their arrangements are consistent with the illustrated atomic structure from the same orientation [see Fig. 1(d)]. The corresponding intensity profile measured along the red dot line in Fig. 1(c) is shown in Fig. 1(e). The average distance between the two Gd atomic columns was 0.36 nm [Fig. 1(e)], which is consistent with the expected distance in Fig. 1(d). These results unambiguously confirm the monoclinic symmetry of the synthesized Gd_2_O_3_.

**Fig. 2:**
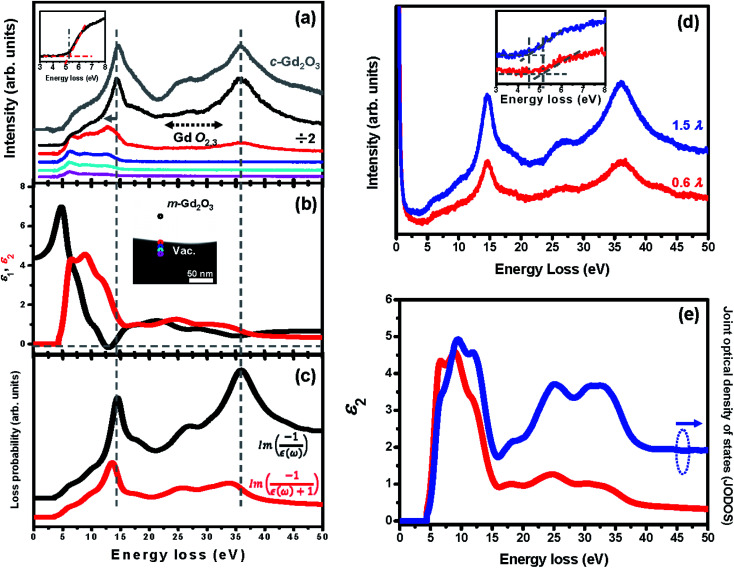
(a) Aloof beam low-loss STEM-EEL spectra acquired from m-Gd_2_O_3_ with the electron probe positioned at the locations marked in HAADF STEM image [middle upper inset in (b)]. The color circles denote probe positions in the inset. Corresponding EEL spectra are shown by the same colors. The low-loss EEL spectrum of c-Gd_2_O_3_ is presented for comparison in grey. (b) The real (black) and imaginary (red) part of the dielectric permittivity of m-Gd_2_O_3_ derived from the black spectrum in (a). Inset is the HAADF STEM image with locations marked for aloof low-loss STEM-EEL spectra. (c) The black and red curves represent the volume and surface energy loss function, respectively. (d) The EEL spectra acquired from m-Gd_2_O_3_ with different thicknesses. Inset in (d) shows EEL spectra redrawn for illustration of bandgap measurement. (e) The imaginary part of dielectric permittivity, *ε*_2_, and the calculated joint optical density of states (JODOS).

While performing EELS in the STEM mode (STEM-EELS), it is possible to preserve the spatial resolution and operate with nonpenetrating electron beam setups when the electron probe is sequentially positioned at the different positions along a chosen direction from the material interior to vacuum. Signal delocalization enables in this case the acquisition of the EELS spectra even when the electron beam is located at 2 nm or farther from the grain surface. Such an aloof setup can eliminate knock-on damage and significantly reduce the beam-induced ionization damage.^18,25^Fig. 2(a) shows the EELS spectra of m-Gd_2_O_3_ recorded using the aloof beam at the different positions from the material interior to vacuum, as indicated by the circles in the HAADF STEM image inset in Fig. 2(a). The low-loss EELS spectrum of c-Gd_2_O_3_ (gray curve) is also shown in Fig. 2(a) for comparison. When the electron probe was positioned in the interior of the material [black circle in the HAADF STEM image inset in Fig. 2(a)], the most intensive spectrum exhibiting a predominantly volume character was obtained [a black curve in HAADF STEM image inset in Fig. 2(a)]. The optical bandgap of about 5.1 eV was determined using the linear fitting method.^26^ No additional features were found in the bandgap region [see inset in Fig. 2(a)]. The energies of the broad excitation features at about 7, 10.2, 14.5, 17.8, 21.6, 27.5, 31.5, and 36 eV energy loss were determined by applying the Lorentz fitting to the EELS spectrum.^19^ The spectra in Fig. 2(a) reveal differences between m-Gd_2_O_3_ and c-Gd_2_O_3_. At first, the two spectral features at 7 eV and 10.2 eV energy loss were observed in m-Gd_2_O_3_ while only a broad feature at 9.2 eV energy loss was found in c-Gd_2_O_3_. In the second, a broad hump between 15 eV and 18 eV energy loss observed in m-Gd_2_O_3_ was absent in c-Gd_2_O_3_. In the third, distinct differences between m-Gd_2_O_3_ and c-Gd_2_O_3_ were found for the Gd O_2,3_-edge between 21 eV and 28 eV energy loss. Since the *C*2/*m* space group of m-Gd_2_O_3_ has a lower degree of symmetry than the *Ia*3̄ space group of c-Gd_2_O_3_; a rather distorted octahedral environment in m-Gd_2_O_3_ would result in more complex bonding and band structures, and exhibit more excitation probabilities for excitonic and/or interband-transitions as compared to c-Gd_2_O_3_.^14^

The single-scattering intensity of the low-loss EEL spectrum dominated by the collective excitations of valence electrons is proportional, to a first approximation, to the imaginary part of the energy loss function, 
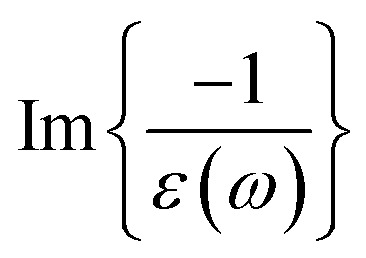
, if one excludes minor effects such as surface scattering, retardation effects, and instrumental broadening.^18,20,25^ After retrieving the real part of the energy loss function, 
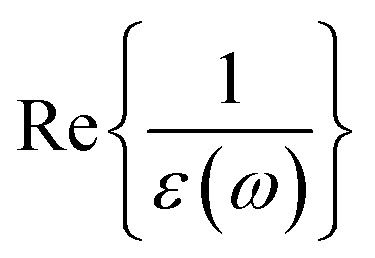
, the real and imaginary parts of the frequency (*ω*)-dependent dielectric function, [*ε*(*ω*) = *ε*_1_(*ω*) + i*ε*_2_(*ω*)], can be calculated *via* the KKA.^18,25^ Thus, the low-loss EEL spectrum can be interpreted in terms of the complex dielectric function of the materials. To facilitate an understanding of the electronic excitations in m-Gd_2_O_3_, one should measure *ε*(*ω*) covering a range of at least up to 50 eV energy loss. The *ε*(*ω*) of m-Gd_2_O_3_ [Fig. 2(b)] was derived by performing the KKA from the black curve in Fig. 2(a). From the *ε*_1_ curve in Fig. 2(b), *ε*_1_ passes through zero with a positive slope at about 13.9 eV and a vanishing *ε*_2_ value of 1.39, leading to a maximum of the energy loss function ∝ 
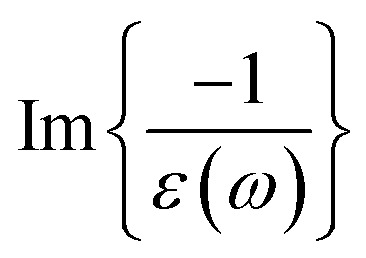
 at 14.5 eV [a black curve in Fig. 2(c)]. This suggests that the peak experimentally observed at 14.5 eV energy loss can be interpreted as a volume plasmon (VP). This value is close to 14.6–15 eV, which was reported earlier for VP in Gd_2_O_3_.^14,18^ The energy of the volume plasma resonance, ℏ*ω*_p_, for the particular excitonic system can be approximated as follows.^18^

1 ℏ*ω*_p_ = [(ℏ*ω*^f^_p_)/*E*_g_^2^]^0.5^

where 
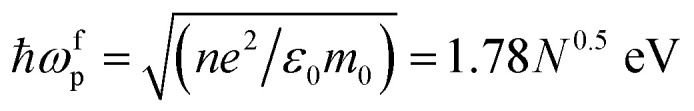
 is the quasi-free electron plasma resonance energy, *E*_g_ = 5.1 eV is the optical bandgap, *n* = *N*/*V* is the electron density, *e* is the electron charge, *m*_0_ is the electron mass, *ε*_0_ is the permittivity of vacuum, *N* is the number of involved valence electrons per cell of m-Gd_2_O_3_, and *V* = 0.436 nm^3^ is the cell volume.^27^ From eqn (1), ℏ*ω*^f^_p_ is 13.57 eV, and *N* is 58.25 electrons per cell or only about 4.9 electrons per Gd atom. This is in line with the density functional theory (DFT) calculations of the m-Gd_2_O_3_ band structure and density of states (DOS),^28^ and the experimental joint optical density of states (JODOS), *J*_1_(*E*) = *Eε*_2_(*ω*)/0.5π(ℏ*ω*_p_^f^)^2^ (ref. 18 and 29) [see Fig. 2(e)]. The plasma resonance here is excited primarily only by less than 5 of 7 quasi-free 4f Gd valence electrons. Semi-local DFT calculations tend to severely underestimate the bandgap of m-Gd_2_O_3_ (2.9 eV instead of 5.1 eV from the experiment) that could be caused by polaronic effects. Shifting the bandgap onset to 5.1 eV qualitatively correlates with the strong JODOS increase that can be tentatively assigned to the first conduction band rising above the bandgap with the local maxima at about 6.5, 9.5, and 11.9 eV energy loss induced mainly by Gd 4f electron states [Fig. 2(e)].^14^ The spectral features at about 7, 10.2, 17.8, 27.5, 31.5, and 36 eV energy loss, that are close to the absorption (*ε*_2_) features at about 6, 8.5, 17.7, 24.1, 29.9, and 33.2 eV energy loss [Fig. 2(b)], may arise from the contributions of interband transitions and/or outer shell ionizations. Indeed, the spectral features at 7, 11, 17.5, and 27.5 eV energy loss have been reported for the excitations from O 2p to Gd 5d orbital electrons, O 2p orbital electrons, and for the Gd O_2,3_ ionization edge arising due to the excitation of Gd 5p electrons.^14^ The broad intensive spectral feature at about 36 eV energy loss cannot be interpreted as a VP because *ε*_1_ does not cross zero about 35 eV [Fig. 2(b)] unlike the interpretation of VPs suggested in the literature.^15,16^ An assignment of this spectral peak will be discussed in more detail below. Fig. 2(d) shows the experimental EEL spectra recorded for various thicknesses at the accelerating voltage of 200 kV. The experimental EEL spectra indicate that both the VP at 14.5 eV and the peak at 36 eV energy loss increased their oscillation strength by increasing the thickness from 0.6*λ* to 1.5*λ* (*λ* is the inelastic mean free path. The log-ratio (relative) method was considered to measure the sample thickness using the DigitalMicrograph software package). The enlarged low-loss EEL spectra are redrawn [see inset in Fig. 2(d)] to illustrate the bandgap measurements.

Cherenkov radiation (CR) can be excited when the material has high dielectric constant or refractive index (*n*), and the TEM accelerating voltage is high enough to induce significant relativistic effects. The differential scattering cross-section for the volume losses including CR losses is described by the following equation.^19,21,26,30,31^2
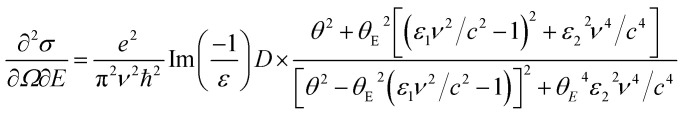
where *ε*(*ω*) = *ε*_1_(*ω*) + i*ε*_2_(*ω*) is the complex dielectric function, *Ω* is the solid angle of scattering, *E* is the energy loss, *D* is the thickness of the specimen in units of the mean free path length for inelastic scattering, *θ*_E_ = *E*/2*γT* is the characteristic scattering angle, *ν* is the velocity of incident electrons, and *c* is the speed of light. In this study, the *ε*_1_ value in m-Gd_2_O_3_ was varied from 4.5 at *ω* → 0 to 7 at the energy loss ≤5 eV [see Fig. 2(b)]. The accelerating voltage used for EELS measurement was 200 kV, yielding *ν* of about 0.7*c*. The conditions satisfied the CR excitation criterion of (*ν*/*c*)^2^*ε* > 1.^19,21,23,30,31^ The CR excitation could therefore appear as a broaden feature at the energy loss ≤5 eV, and affect the KKA results and determination of the bandgap energy. In addition, the more intensive CR excitation generated with increasing specimen thickness tended to shift of the bandgap toward lower energies from 5.1 eV to 4.9 eV [see Fig. 2(c)].

**Fig. 3:**
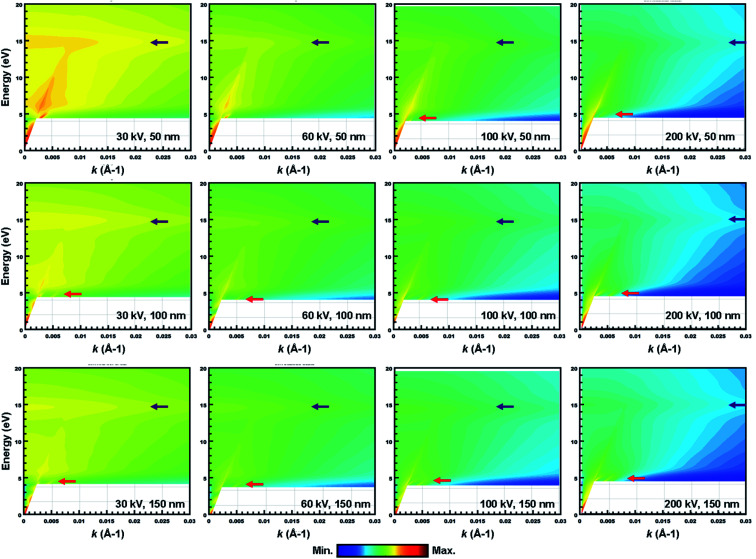
The calculated relativistic *E*–*k* maps (*E* is energy loss and *k* is momentum transfer) for m-Gd_2_O_3_ slabs with thicknesses of 50 nm (top), 100 nm (middle), and 150 nm (bottom panel) at accelerating voltages of 30 kV, 60 kV, 100 kV, and 200 kV.

**Fig. 4:**
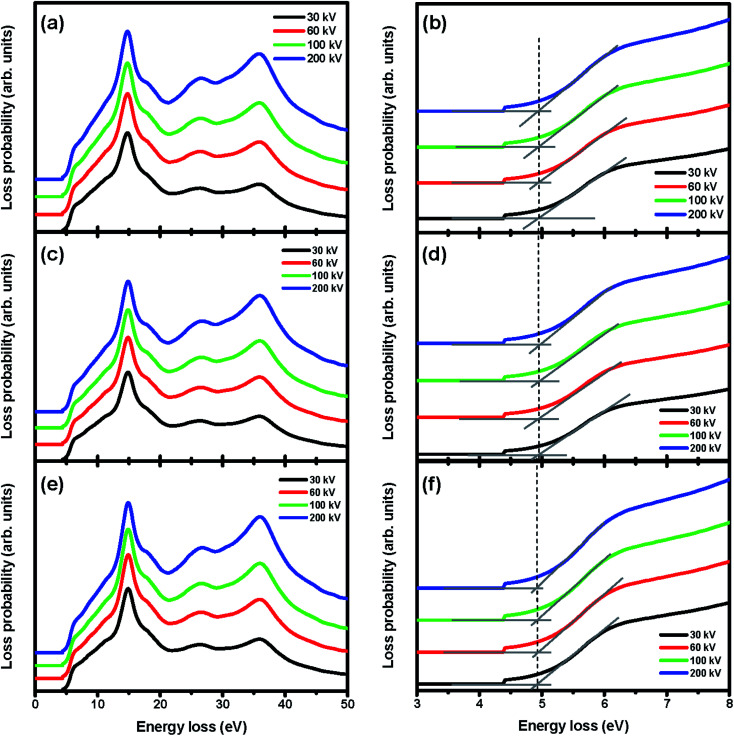
The calculated relativistic loss probabilities of m-Gd_2_O_3_ slab per unit electron path length integrated over *k* in the range of 0–00.3 Å^−1^ for the thickness for (a) 50 nm, (c) 100 nm, and (e) 150 nm. The same EEL spectra are redrawn in (b), (d) and (f) for illustrating the bandgap measurements.

To evaluate the CR effect, we first calculated the relativistic energy *versus*-momentum (*E*–*k* maps) for Gd_2_O_3_ slabs of 50 nm, 100 nm, and 150 nm in thickness at different accelerating voltages varying from 30 kV to 200 kV, respectively, which were calculated using Kröger's equation,^21^ as shown in Fig. 3. From the calculated *E*–*k* maps, the VP at 14.5 eV (marked by the purple arrows) enhanced its intensity by increasing both the accelerating voltages and thickness, thus indicating its nondispersive character. For the 50 nm-thick m-Gd_2_O_3_ slab, CR excitation showed distinct dispersion features near the bandgap onset below 5 eV (marked by red arrows) when the accelerating voltage was 100 kV. For m-Gd_2_O_3_ slabs of 100 nm and 150 nm in thickness, the CR excitations displayed dispersion features near the bandgap onset below 5 eV (marked by red arrows) at all the accelerating voltages. The related relativistic loss probabilities per unit electron path length along the electron trajectory and integrated over the *k* range up to 0.03 Å^−1^ are shown in Fig. 4. Fig. 4(a), (c), and (e) present thickness-dependent EELS spectra calculated for different accelerating voltages. Similar to Fig. 3, the VP at 14.5 eV and the peak at 36 eV energy loss were observed regardless of the chosen thicknesses and used the accelerating voltages. However, the oscillation strength of the peak at 36 eV energy loss increased with the increasing accelerating voltages in agreement with the report.^32^ The same EEL spectra shown in Fig. 4(a), (c), and (e) are redrawn in Fig. 4(b), (d), and (f) to illustrate the bandgap measurements. With increasing specimen thickness and accelerating voltages, the generated CR tended to shift the bandgap toward lower energies from about 4.95 eV to 4.9 eV. The discrepancy in the bandgap measurements between the experimental spectra (Fig. 2) and the calculated spectra (Fig. 4) could be due to different integrated *k* ranges. This will be discussed in more detail in Fig. 7. Thus, to minimize the CR effect, it is recommended to reduce the accelerating voltage to less than 60 kV.^20,31^ Based on our calculations, the accelerating voltage should be less than 30 kV, when *ν* is about 0.33 c, to satisfy the condition (*ν*/*c*)^2^*ε*_1_ < 1. However, decreasing the accelerating voltage to 30 kV is practically difficult due to the limitations of available high voltage settings, stability, and tedious alignments in both TEM and Gatan GIF systems needed for such changes. Thus, the EEL spectra in Fig. 2 and 7 were recorded with a sufficient thickness of 50 nm to suppress the surface excitations by analyzing the material interior^33^ and minimizing the CR excitation in the spectral region ≤5 eV. Furthermore, the KKA results in Fig. 2(b) were carefully processed to remove the zero-loss peak and iteratively remove the surface losses and other retarding effects as described in the literature.^20^

To investigate surface-related resonances such as surface plasmons (SPs), the aloof electron beam was continuously positioned at the specimen edge at the grazing incidence and a few nanometers away from the specimen edge. The red spectrum in Fig. 2(a) obtained at the grazing incidence at the specimen edge, which shows that the VP peak was redshifted from about 14.5 eV to 13 eV energy loss and the intensity of the spectral feature at about 36 eV energy loss was significantly decreased. The spectral peak at about 13 eV energy loss can be interpreted as an SP because the small negative values of *ε*_1_ in this energy range lead to a maximum in the energy loss function ∝ 
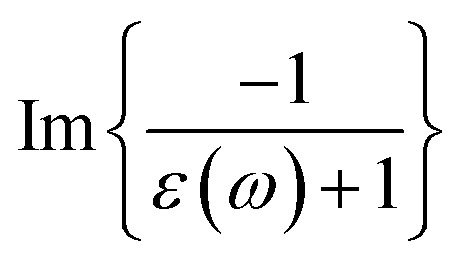
 at 13 eV [see the red spectrum in Fig. 2(c)].^13,14,18,25^ After moving the electron beam from the specimen edge into the vacuum [*e.g.*, see the blue, cyan, and pink spectra in Fig. 2(a)], the oscillation strength of the SP at about 13 eV decreased with the distances, indicating the presence of evanescent wave fields of SPs.^13,14,18,25^ Interestingly, the broad shoulder at about 7 and 10.2 eV energy loss decreased much slower than the SP peak at 13 eV energy loss, and further showed a prominent spectral onset at about 7 eV energy loss [*e.g.*, see the pink spectrum in Fig. 2(a)], confirming the surface character of the related excitations. The interband transitions generating weakly bound delocalized excitons presumably of the Wannier–Mott type readily build up collective resonances at the material surface. Furthermore, the surface resonances associated with transverse excitonic onsets could be assigned to surface exciton polaritons (SEPs) if the condition *ε*_2_ > |*ε*_1_| ≥ 0 is fulfilled.^13,14,17^ Indeed, the excitonic and/or interband transitions from the O 2p to the Gd 5d states can contribute to the spectra in the low-loss range from 7 to 11 eV,^14^ corresponding to the strong JODOS bands at about 6.5, 9.5, and 11.9 eV energy loss [Fig. 2(d)]. Therefore, it is reasonable to suggest that the spectral features at about 7 eV and 10.2 eV energy loss can be interpreted as SEPs.

**Fig. 5:**
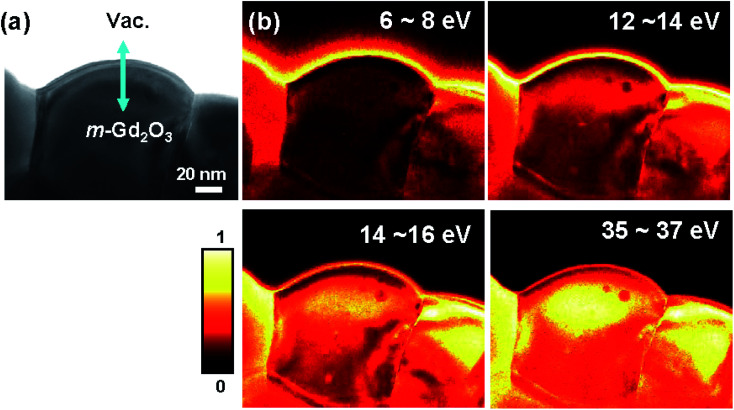
(a) EFTEM zero-loss image. (b) The EFTEM-SI intensity maps acquired at energy losses of 7 ± 1 eV, 13 ± 1 eV, 15 ± 1 eV, and 36 ± 1 eV. The color scale bar represents the linearly normalized image intensity.

The interband transitions and plasmon losses can be visualized in real space using EFTEM-SI in the selected specific energy loss range.^14,17,18^ EFTEM-SI was performed to examine the SEP, SP, and VP excitations in m-Gd_2_O_3_ with a 2 eV energy window centered at 7, 13, 15 eV, and 36 eV energy loss [see in Fig. 5(b)], where the related spectral features were found. Fig. 5(a) shows the corresponding zero-loss TEM image of the oxide area where EFTEM-SI analyses were conducted. The intensity maximum in the EFTEM SI image representing the spatial location of the SEP at about 7 eV and the SP at 13 eV energy loss evidently visualizes the related surface excitations at the edge of an oxide grain with the evanescent wave field decaying into the vacuum. In contrast, the intensity maximum of the VP at about 14.5 eV energy loss and of the broad band at about 36 eV energy loss was strongly localized within the bulk material interior, thus unambiguously indicating the volume character of the excitations.

**Fig. 6:**
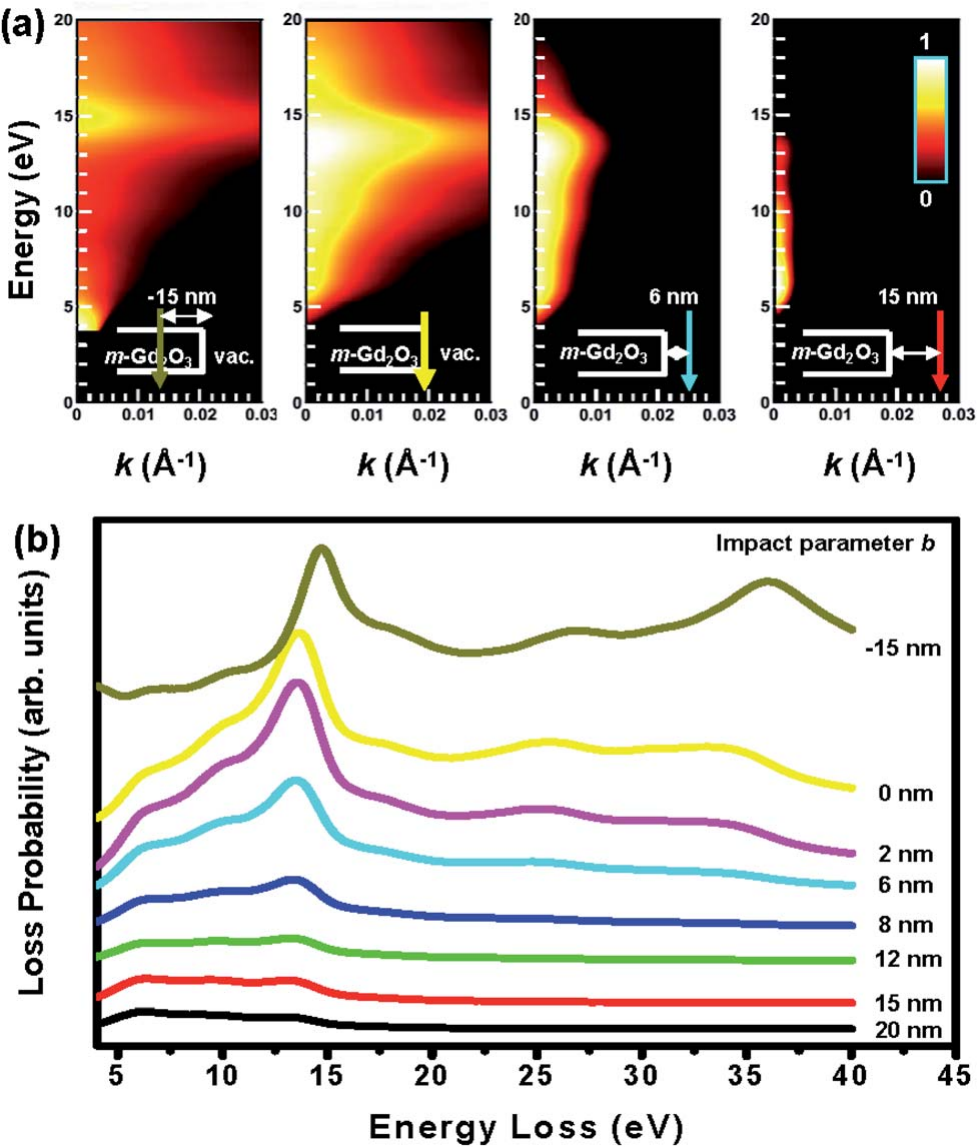
(a) The calculated relativistic *E*–*k* intensity maps for 50 nm-thick m-Gd_2_O_3_ slab with impact parameters *b* = −15, 0, 6, and 15 nm. The electron probe position exploited for the calculations is depicted in each corresponding inset. (b) The calculated relativistic loss probabilities of 50 nm-thick m-Gd_2_O_3_ slab per unit electron path length integrated over *k* in the 0–00.3 Å^−1^ range with impact parameters *b* = −15, 0, 2, 6, 8, 12, 15, and 20 nm.

To gain deeper physical insights into the aloof STEM-EEL spectra shown in Fig. 2(a) as a function of impact parameter *b*, the relativistic *E*–*k* maps^22,23^ were calculated for a 50 nm thick m-Gd_2_O_3_ layer and shown in Fig. 6(a). Fig. 6(b) shows related relativistic loss probabilities per unit electron path length along the electron trajectory and integrated over the *k* range up to 0.03 Å^−1^. The calculated *E*–*k* maps in Fig. 6 reveal the predominant VP at about 14.5 eV energy loss when the electron probe was positioned inside the slab at *b* = −15 nm. For the electron probe sequentially located at the edge in a grazing incidence (*b* = 0 nm) at *b* = 6 and 15 nm away from the edge, the SP at about 13 eV energy loss was initially greatly enhanced at the *b* = 0 nm and then its oscillation strength decreased with increasing *b* values, thus indicating its surface character in the presence of evanescent wave fields. The calculations also successfully reproduced the SEPs at about 7 eV and 10.2 eV energy loss at *b* ≥ 12 nm. Both calculated *E*–*k* maps in Fig. 6(a) and corresponding STEM-EELS spectra in Fig. 6(b) appear in good agreement with the experimental results presented in Fig. 2(a).

As far as the spectral feature at about 36 eV energy loss are concerned [the red spectrum in Fig. 2(a)], this peak appeared at the specimen edge and then increased its oscillation strength with increasing specimen thickness [see Fig. 2(c)]. Most importantly, contrary to the SP at 13 eV and the VP at 14.5 eV energy loss, this peak did not shift while the probe moved from the edge to the material interior. Furthermore, the peak also decayed into the vacuum at about 6 nm from the edge measured from the intensity profile in the corresponding EFTEM-SI image [Fig. 5(b)]. This was consistent with the calculated relativistic loss probabilities spectra for *b* = 6 nm [Fig. 6(b)]. Interestingly, the decay length of about 6 nm is longer than the delocalization of about 1.5 nm calculated using the formula, 0.5*λ*/(*θ*_E_^3/4^), where *λ* is the wavelength and *θ*_E_ is the relativistic characteristic angle,^18^ implying that the 36 eV peak might be associated with both bulk and surface excitations. Indeed, the onset of the peak at about 36 eV energy loss closely correlates with the broad *ε*_2_ feature at about 33.2 eV energy loss and the corresponding oscillating *ε*_1_ structure [Fig. 2(b)]. This *ε*_2_ feature signifies the diffused oscillator strengths induced by the bulk transverse interband transitions from deep 5p states to 5d bands,^14^ which are related to a broad feature at the same energy loss in the JODOS [Fig. 2(d)]. Since the criteria condition of *ε*_2_ > |*ε*_1_| ≥ 0 discussed above is fulfilled, this spectral feature can also be interpreted as the excitation of SEPs in m-Gd_2_O_3_.

**Fig. 7:**
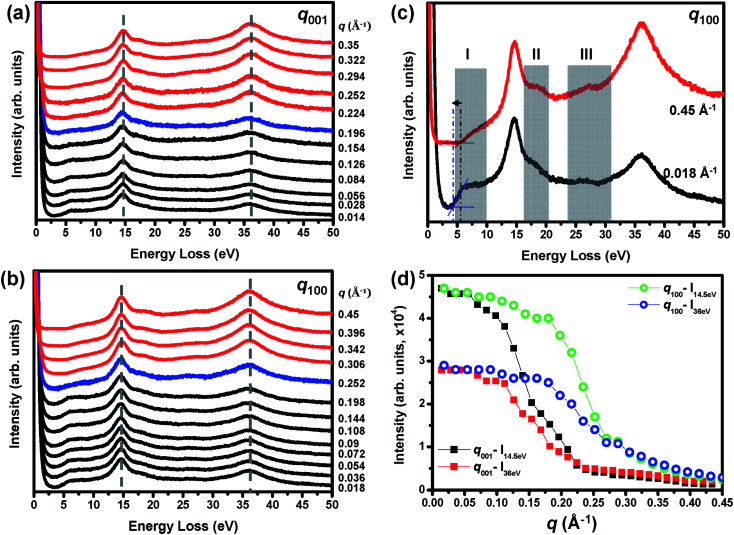
Momentum (*q*)-dependent EEL spectra collected with the *q* parallel to the (a) [001] and (b) [100] directions. The spectra are all normalized to the intensity of the peak at 14.5 eV and then displaced vertically to improve readability. (c) The enlarged *q*-dependent EEL spectra at *q* = 0.018 Å^−1^ and 0.45 Å^−1^ from (b). See the detailed description for shadowed regions I, II, and III in the main text. (d) The momentum (*q*) dependency of oscillator strengths of the VP at 14.5 eV and the SEP at 36 eV energy loss along the [001] and [100] directions.

The momentum (*q*)-dependent EELS (*q*-EELS) is a powerful method to examine the excitations in solids varying both the relatively large momentum transfer (Δ*q*) and the energy loss Δ*E*.^18,19,25^Fig. 7(a) and (b) show the *q*-EELS spectra acquired along the [001] and [100] directions up to the Brillouin zone (B. Z.) boundary for *q* values of about 0.35 Å^−1^ and 0.45 Å^−1^, respectively. Fig. 7(c) presents the EEL spectra corresponding to the *q* values of 0.018 Å^−1^ and 0.45 Å^−1^ acquired along the [100] direction to enhance the observed differences. From the spectra in Fig. 7(a)–(c), a few interesting findings have to be pointed out. At first, the VP at about 14.5 eV energy loss displays a nondispersive behavior along the [001] and [100] directions probably due to confining the nearby interband transitions.^18,25^ Meanwhile, the SEP at about 36 eV energy loss also exhibits a nondispersive behavior, resulting from its band structure with a relatively small curvature.^15,18,25^ In the second, the plateau between 5 to 10 eV energy loss was observed [see the black color spectra in Fig. 7(a) and (b)] when the *q* was less than 0.2 Å^−1^ and then increased the oscillator strengths to enhance the SEP features at about 7 eV and 10.2 eV energy loss when the *q* was larger than 0.2 Å^−1^ [see the red color spectra in Fig. 7(a) and (b) and see the shadowed region I in Fig. 7(c)]. In the third, the measured bandgap energy varied from about 4.4 eV to 5.4 eV. In the fourth, the spectral features at about 17.8 eV energy loss and the Gd O_2,3_-edge (21 to 28 eV energy loss) enhanced their oscillator strengths with increasing *q* values [see shadowed regions II and III in Fig. 7(c)]. Finally, the oscillation strength of the SEP at about 36 eV energy loss increased with increasing *q* values and became more intensive than the VP peak at *q* > 0.25 Å^−1^ as one can see on comparing the black and red spectra in Fig. 7(a)–(c).

In general, the excitation probability 
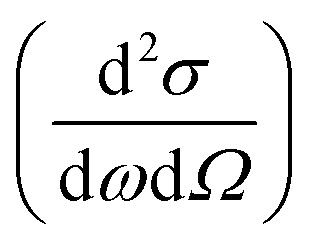
 is inversely proportional to *q*^2^, according to the following the equation:^18,25^3
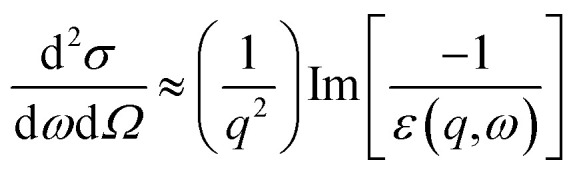
where *q* is momentum transfer. From eqn (3), the excitation probability should decrease with increasing *q* values, leading to a decrease in the oscillator strengths. In addition, the VP dispersion usually exhibits a parabolic dispersion upward to higher energies at larger *q* values accompanied with decreasing oscillator strengths and peak broadening in terms of the full-width at half maxima (FWHM, Δ*E*_1/2_ = ℏ/*τ*, where ℏ is Planck's constant and *τ* is relaxation time) beyond the cutoff wavevector (*q*_c_).^18,25^ Although both the VP and the SEP at about 36 eV energy loss display the nondispersive behavior with no distinct changes of the Δ*E*_1/2_ [see Fig. 7(a) and (b)], this is hardly reconcilable with a plasmon behavior when the Δ*E*_1/2_ should increase rapidly with *q*.^18,25^ However, Fig. 7(d) shows that the oscillator strengths still decays exponentially as expected and drop at different *q*_c_ values along the [001] and [100] direction, indicating the anisotropy of the electronic structures of m-Gd_2_O_3_. The further inspection of the curves in Fig. 7(d) indicates different dropped slopes for the VP and the SEP at about 36 eV energy loss, suggesting the different damping mechanisms. The plasmons, as collective oscillations of valence band electrons, would induce the kinematically allowed single-electron excitations and then start damping because the plasmons transfer all of their energy to excite single-electron transitions and create the electron–hole pairs when the plasmon wavevector *q* exceeds the *q*_c_ values.^18,25^ On the contrary, the SEP at about 36 eV energy loss may be treated here as an intrinsic characteristic of interband transitions, which is a kind of single-electron excitation. This is because m-Gd_2_O_3_ with a sufficient thickness of 50 nm was used for *q*-EELS measurements to suppress its surface character in the material interior.^29,33^ Thus, it appears that the SEP at about 36 eV energy loss was only damped by the interactions between the excitons. Therefore, the first step needed for the transfer of all the SEP energy to excite a single-electron transition and create an electron–hole pair was absent. This could reasonably explain the different damping rates observed in the study for the VP and the SEP excitations.

## Conclusions

The electronic excitations of valence electrons in monoclinic Gd_2_O_3_ were thoroughly studied using STEM-EELS with the aloof electron beam and electron diffraction to gain both the spatial and momentum resolutions. By positioning the electron probe at the specimen edge in a grazing incidence and in the material interior, the SP at about 13 eV and the VP at 14.5 eV energy loss were observed. Intriguingly, unusual surface-related excitations, SEPs, were observed at about 7, 10.2, and 36 eV energy loss with an evanescent wave field decaying into the vacuum as it was confirmed by EFTEM SI in agreement with the relativistic energy *versus*-momentum (*E*–*k*) maps calculations. The momentum (*q*)-dependent EELS measurements showed that the SEP features at about 7 and 10.2 eV energy loss appeared to be a function of *q* and revealed the nondispersive behavior for both VP at 14.5 eV and SEP at about 36 eV energy loss. Indeed, variations in the critical wavevector *q*_c_ were observed in different *q* directions, indicating the anisotropy of the electronic structure of monoclinic Gd_2_O_3_.

## Conflicts of interest

There are no conflicts of interest to declare.

## Supplementary Material
